# Supplementary material for: Functional Decline in the Cancer Patient: A Review
Source: Cancers (Basel). 2022 Mar 8;14(6):1368. doi: 10.3390/cancers14061368 (PMC8946657; doi:10.3390/cancers14061368)
Supplement: Supplementary file 1 [file cancers-14-01368-s001.zip › cancers-1543641-supplementary.pdf]

cancers

Review

Functional Decline in the Cancer Patient: A Review

Jaidyn Muhandiramge, Suzanne G. Orchard, Erica T. Warner, G. J. van Londen and John R. Zalcberg

Supplementary Material: Literature search strategy

Databases searched: Google Scholar, PubMed, OVID Medline

Search strategy:

An initial scoping literature search was conducted using Google Scholar and PubMed using the search terms cancer; functional decline; activities of daily living. This search was used to gauge the availability of literature and identify landmark studies investigating the link between cancer and functional decline

A more comprehensive search was conducted using OVID Medline (9 October 2021). The search terms used can be found below.

Search term	Search type
Functional decline OR functional status OR activity of daily living OR activities of daily living AND;	Title
Cancer	Title

The search yielded a total of 195 articles.

Additional screening:

Articles were title screened and included on the basis of being relevant to one of the following topics:

- The link between cancer/cancer treatment and functional decline
- Assessment of functional decline in cancer survivors
- Strategies to prevent or manage functional decline in cancer survivors
- Clinical implications of functional decline in cancer survivors including increases in mortality or decreases in quality of life

Articles were only included if they focussed on an older adult population; studies in paediatric or young-to-middle-aged adults were excluded. Non-English language articles were excluded.

A total of 154 articles remained after title screening. All articles were sorted into four groups: evidence linking cancer and functional decline, assessment of functional decline, prevention and management of functional decline, and clinical implications of functional decline.

Articles were subsequently abstract screened, with high quality articles being selected for inclusion in this review. Features used to identify high quality articles included large sample size, presence of a control group, separation and adjustment for treatment factors, long follow up time, and multi-site or national/international recruitment.
